# Appointment of Dr. Baxley, of Baltimore

**Published:** 1850-10

**Authors:** 


Dr. Baxley of Baltimore has received and accepted the appointment of Professor of Anatomy in the Medical College of Ohio, to fill the vacancy created by the death of Dr. Shotwell. All the chairs in this Institution are now full, presenting an able and accomplished corps of teachers.

